# Authors' Reply: Measurement Errors in Schizophrenia Epidemiology

**DOI:** 10.1371/journal.pmed.0020300

**Published:** 2005-09-27

**Authors:** John McGrath, Sukanta Saha, Joy Welham, David Charles Chant

**Affiliations:** **1**University of QueenslandWacol, QueenslandAustralia; **2**Queensland Centre for Mental Health ResearchWacol, QueenslandAustralia

The letter from Hambidge highlights the heterogeneous nature of schizophrenia [1]. In order to diagnose schizophrenia, modern diagnostic criteria require the exclusion of other general somatic conditions that can mimic psychotic symptoms. Compliance with screening protocols designed to identify these disorders varies widely, even in developed countries. We agree with the correspondent that some studies included in our recent systematic review [2] would have probably included individuals who were subsequently found to have “secondary schizophrenia” (i.e., false positives). Thus, this issue would slightly inflate the prevalence estimate. The inappropriate inclusion of false positives is only one of a very long list of methodological factors that contribute to imprecision in the estimation of the incidence and prevalence of schizophrenia. The critical issue for the research community is how best to partition out measurement error from “true” variations in the incidence or prevalence of schizophrenia. In the absence of more refined phenotypes for the many different disorders that contribute to the syndrome of schizophrenia (e.g., by the use of yet-to-be-identified biomarkers), standard epidemiological studies of the incidence and prevalence of schizophrenia may have reached their limits of precision.
